# Coverage maps demonstrate 3D Chopart joint subluxation in weightbearing CT of progressive collapsing foot deformity

**DOI:** 10.1038/s41598-022-23638-3

**Published:** 2022-11-12

**Authors:** Andrew Behrens, Kevin Dibbern, Matthieu Lalevée, Kepler Alencar Mendes de Carvalho, Francois Lintz, Nacime Salomao Barbachan Mansur, Cesar de Cesar Netto

**Affiliations:** 1 Department of Orthopedics and Rehabilitation, University of Iowa, 200 Hawkins Drive, Iowa City, IA 52242 USA; 2 Service d’orthopédie Traumatologie, Centre Hospitalier Universitaire de Rouen, 37 Boulevard Gambetta, 76000 Rouen, France; 3 Clinique de L’Union, Bd Ratalens, 31240 Saint-Jean, France; 4 Department of Orthopedics and Traumatology, Escola Paulista de Medicina UNIFESP, São Paulo, SP Brazil

**Keywords:** Musculoskeletal system, Bone imaging, Three-dimensional imaging, Tomography

## Abstract

A key element of the peritalar subluxation (PTS) seen in progressive collapsing foot deformity (PCFD) occurs through the transverse tarsal joint complex. However, the normal and pathological relations of these joints are not well understood. The objective of this study to compare Chopart articular coverages between PCFD patients and controls using weight-bearing computed tomography (WBCT). In this retrospective case control study, 20 patients with PCFD and 20 matched controls were evaluated. Distance and coverage mapping techniques were used to evaluate the talonavicular and calcaneocuboid interfaces. Principal axes were used to divide the talar head into 6 regions (medial/central/lateral and plantar/dorsal) and the calcaneocuboid interface into 4 regions. Repeated selections were performed to evaluate reliability of joint interface identification. Surface selections had high reliability with an ICC > 0.99. Talar head coverage decreases in plantarmedial and dorsalmedial (− 79%, *p* = 0.003 and − 77%, *p* = 0.00004) regions were seen with corresponding increases in plantarlateral and dorsolateral regions (30%, *p* = 0.0003 and 21%, *p* = 0.002) in PCFD. Calcaneocuboid coverage decreased in plantar and medial regions (− 12%, *p* = 0.006 and − 9%, *p* = 0.037) and increased in the lateral region (13%, *p* = 0.002). Significant subluxation occurs across the medial regions of the talar head and the plantar medial regions of the calcaneocuboid joint. Coverage and distance mapping provide a baseline for understanding Chopart joint changes in PCFD under full weightbearing conditions.

## Introduction

The transverse tarsal joint complex (Chopart articulations) is a key element of the peritalar subluxation (PTS) seen in progressive collapsing foot deformity (PCFD)^1^. Through these structures, most of the pathological features associated with PCFD occur^2^. Abduction of the midfoot, medial arch collapse, and forefoot varus may have substantial or minor contributions from the talonavicular and the calcaneocuboid joint^3–5^. As PTS occurs in PCFD, structures distal to an initially fixed talus are expected to deviate dorsolaterally, contributing substantially to the described deformities^6,7^. Prior work attempted to assess these behaviors using various methods like simulated weight-bearing computed tomography (WBCT) and fluoroscopy, finding abduction and eversion but conflicting results regarding plantarflexion through these joints^8–10^.

The recent use of WBCT to evaluate PTS has produced important data to help understand this pathological functioning^11,12^. Using two-dimensional tools in coronal plane imaging, the amount of subluxation at the posterior and middle facets was found to be correlated with PCFD diagnosis and severity^6,11,13,14^. Dibbern et al. performed an objective three-dimensional (3D) WBCT analysis of the subtalar joint in PCFD using 3D distance maps (DMs) and introducing the concept of coverage mapping (CM)^15^. They showed that subluxation of the calcaneus underneath the talus was more prominent in the middle facet than in the posterior facet of the subtalar joint, while simultaneously identifying decreases in interbone distance in the sinus tarsi and subfibular regions, explaining lateral impingements in PCFD^16,17^.

These CM and DM techniques may help improve understanding of bone positioning and interactions through the Chopart complex in PCFD as previous research has not directly assessed the articular interfaces of the talonavicular and calcaneocuboid joints under physiological load^8,9^. Information provided by the 3D mapping specifically related to plantarflexion and subluxation may be of particular value in diagnosing, staging, and estimating treatment impacts in PCFD. Therefore, the objective of this study was to compare distance and coverage map differences between loaded Chopart joints of PCFD and control patients using full weightbearing CT. We hypothesized that a significant amount of decreased articular coverage, indicative of subluxation, would be present in the talonavicular and calcaneocuboid joints in PCFD compared to controls. We further hypothesized that medial widening and lateral narrowing of intra-articular distances would be observed in the talonavicular and calcaneocuboid joints consistent with subluxation. Finally, we sought to understand whether present gold standard methods for selection of articular surfaces are reliable for use in understanding joint interaction.

## Methods

### Design

This retrospective case control study obtained University of Iowa’s institutional review board approval (IRB# 201904825). It complied with the both the Health Insurance Portability and Accountability Act (HIPAA) and the Declaration of Helsinki. Informed consent was obtained from all subjects. This manuscript follows the Strengthening the Reporting of Observational Studies in Epidemiology (STROBE case–control) guidelines (Supplementary material: Appendix)^18^.

### Sample

#### PCFD group

The first 20 patients with a PCFD were selected from a randomized list to have undergone a WBCT at our institution between 2014 and 2021. Adults (over 18 years old) with clinical diagnosis of PCFD were included. Patients presenting with a stage 1 (flexible) class A, B, C, D, or a combination of classes were admitted^19^. Patients were excluded if they were found to have a rigid deformity at physical examination, any prior PCFD surgery or metallic implants deterring visualization of the first and fifth rays. Class E deformities (valgus of the ankle) were also excluded^19^.

#### Control group

A matched control group of 20 feet was selected from adult volunteers that underwent WBCT. Individuals were excluded if they had any hindfoot complaint (current and prior), signs of any deformity or arthritis (hindfoot, midfoot and forefoot) noticeable during imaging assessment. A foot and ankle offset (FAO) bellow 5.2% was required for this group of patients^20^.

### Image acquisition

WBCT acquisition was conducted with patients instructed to bear weight in a natural, upright standing position with feet approximately at shoulder width to distribute weight evenly between their two lower limbs. Studies were performed with a cone-beam computed tomography (CT) scanner (HiRise®, LLC, Warrington, PA, USA).

### Image assessments

#### 3D distance mapping

The 3D boundaries of the talus, calcaneus, navicular, and cuboid were extracted from WBCT images using an automated segmentation protocol (Disior Bonelogic 2.0; Disior Ltd®, Helsinki, Finland). Resulting surfaces were exported as triangulated surface models to Geomagic Design X (3D Systems). Articular facets were selected in Geomagic Design X in two separate trials by the same reader^15^.

**Figure 1:**
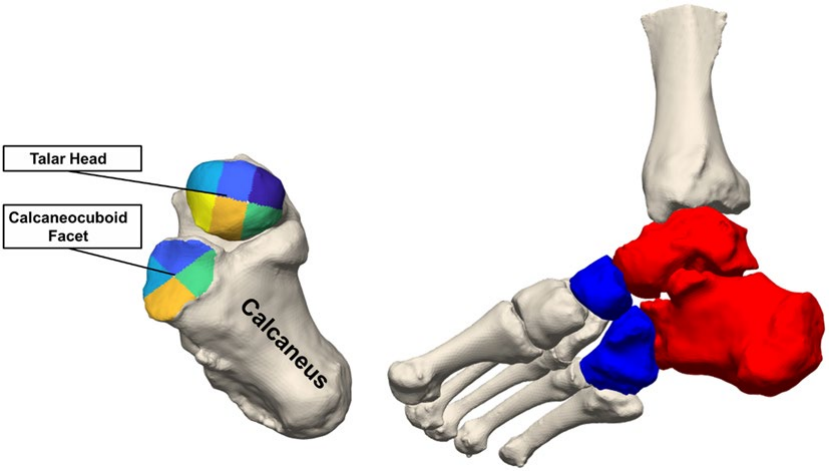
Chopart joint articulations were analyzed with respect to the talus and the calcaneus. The talar head was divided into 6 regions and the calcaneocuboid facet was divided into 4 regions using the principle axes of the joint surface (Left). The Chopart joint occurs between the red (talus and calcaneus) and blue (navicular and cuboid) bones (Right).

Distance measurements were performed along the talar head (articular surface for the navicular) and the articular surface for the cuboid on the calcaneus. Detailed regional analysis was conducted by dividing the talar head into six subregions (Fig. 1) using the principal axes of the joint surface^21^. The cuboid facet of the calcaneus was similarly divided into quadrants: medial, superior, lateral, and inferior. Measurements performed in articular areas were defined using the distance along the normal direction of vectors projected from the subchondral bone of the hindfoot (talar head and calcaneocuboid facet) to their respective midfoot counterparts (the posterior facets of the navicular and cuboid).

#### Coverage maps

Coverage percent was calculated by finding both the total area of each division and the total area of the division that was covered. The area covered divided by the total area of the division yields the coverage percent for that division. We defined coverage as anywhere on the joint that had a joint space width (JSW) of less than five millimeters. We chose a threshold of five millimeters in order to capture all possible parts of the joint that could be considered covered. Lintz et al., reports distances of approximately two millimeters in the talonavicular joint^22^. Other previous studies have used thresholds of four millimeters in joints in the foot^23^. We wanted to ensure adequate characterization of coverage in the joint; the most prudent way to do so was to raise the coverage threshold by one millimeter. Since the method of distance measurement was based on the normal vectors, most regions that were uncovered were defined as such due to the lack of intersection of the normal vector with the opposing bone, not due to being greater than the five-millimeter threshold.

**Figure 2:**
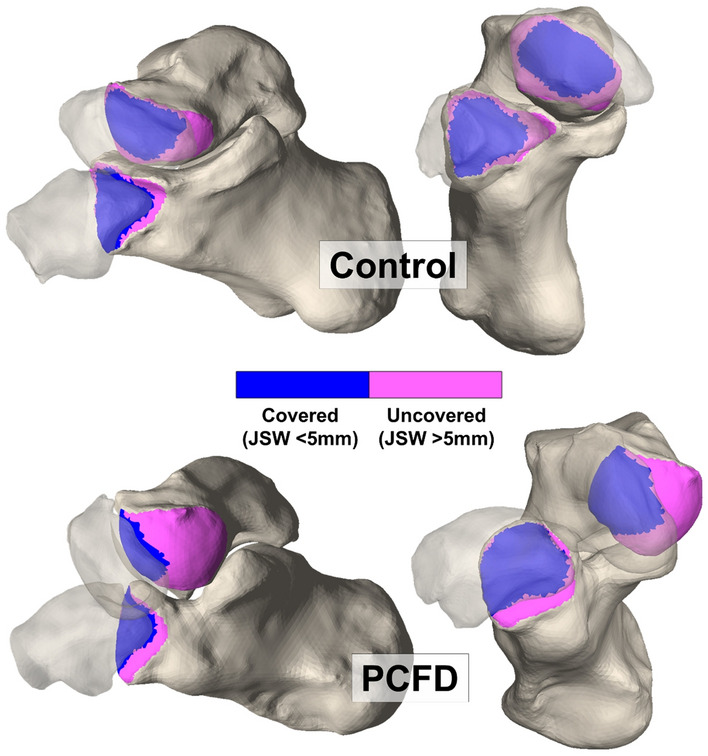
Medial and anterior views of the Chopart joint in representative coverage maps of control and Progressive Collapsing Foot Deformity feet. PCFD tended toward decreased medial coverage and increased lateral coverage in both the calcaneocuboid and talonavicular joints.

Colored CMs were created to assess coverage on the talar head and the calcaneocuboid (CC) facet^15^. Pink was chosen to highlight uncoverage of articular regions, as a result of the overall 3D deformity in PCFD, that were either completely uncovered or had distances greater than 5 mm. Blue indicated coverage of the joint with less than 5 mm distance between bones (Fig. 2).

The talonavicular coverage angle (TNCA) was obtained using the segmentation and the automatic angle tool based on two-dimensional (2D) projections of 3D axes^24^. Finally, a repeatability study was performed to quantify variability in articular selections on the calcaneus and talar head. Models were recreated and the joint area was selected on each bone.

### Statistical analysis

Statistical calculations were performed in MATLAB and Excel using Visual Basic for Applications. Normality was determined using the Shapiro–Wilk Test. *P* values for data sets that were normally distributed were calculated using a two-tailed Student’s T-test; *P* values for data sets that were not normally distributed were calculated using the Mann–Whitney-Wilcoxon U test. Pearson correlation was utilized for correlation between variables. Intraclass correlation coefficients were computed to evaluate interrater selections of the articular areas.

### Ethics and means of dissemination

This work was conducted under University of Iowa’s institutional review board approval (IRB# 201904825). Subjects signed an informed consent prior to inclusion.

### Transparency declaration

The author affirms that this manuscript is an accurate, honest, and transparent account of the study reported; that no important aspects of the study were omitted; and that any discrepancies from the study as planned (and, if relevant, registered) were carefully explained.

## Results

No significant differences were found in the patient characteristics between PCFD and control groups with respect to age (*P* = 0.80), sex (*P* = 1.00), and BMI distributions (*P* = 0.40) (Table 1).

**Table 1:** Patient demographics of progressive collapsing flatfoot deformity and control patients.

Characteristic	Control (n = 20)	PCFD (n = 20)	*P* value
Male, No	6	6	–
Female, No	14	14	–
Age, mean ± SD, y	48.0 ± 19.9	49.5 ± 17.6	0.800
BMI, mean ± SD, kg/m^2^	30.3 ± 8.7	32.7 ± 8.0	0.397

*BMI* body mass index, *PCFD* progressive collapsing foot deformity, – no entries.

### Coverage maps

**Figure 3:**
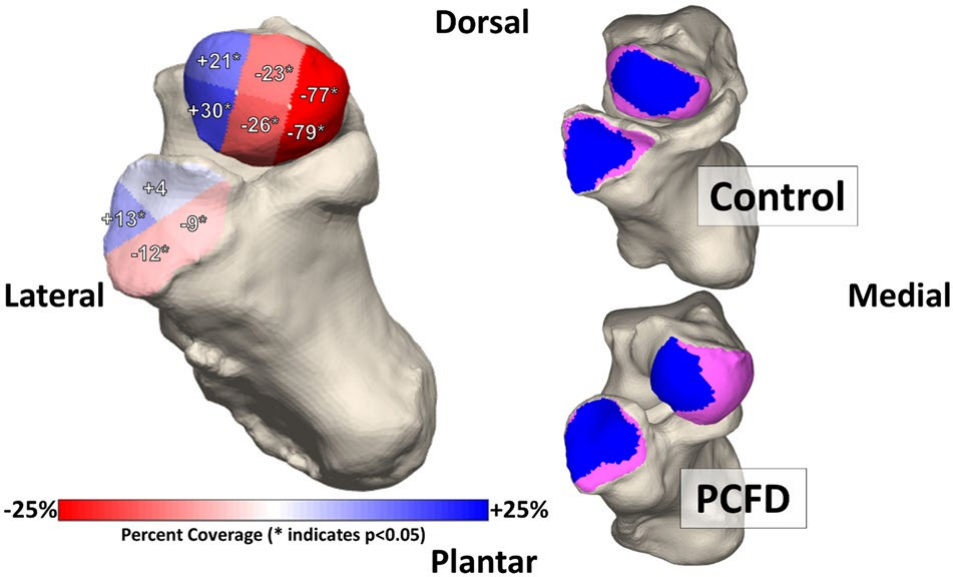
Average percent differences in coverage for the 6 talar head and 4 calcaneocuboid facet regions. Red represents a decrease in coverage from controls to Progressive Collapsing Foot Deformity patients while blue represents an increase.

**Figure 4:**
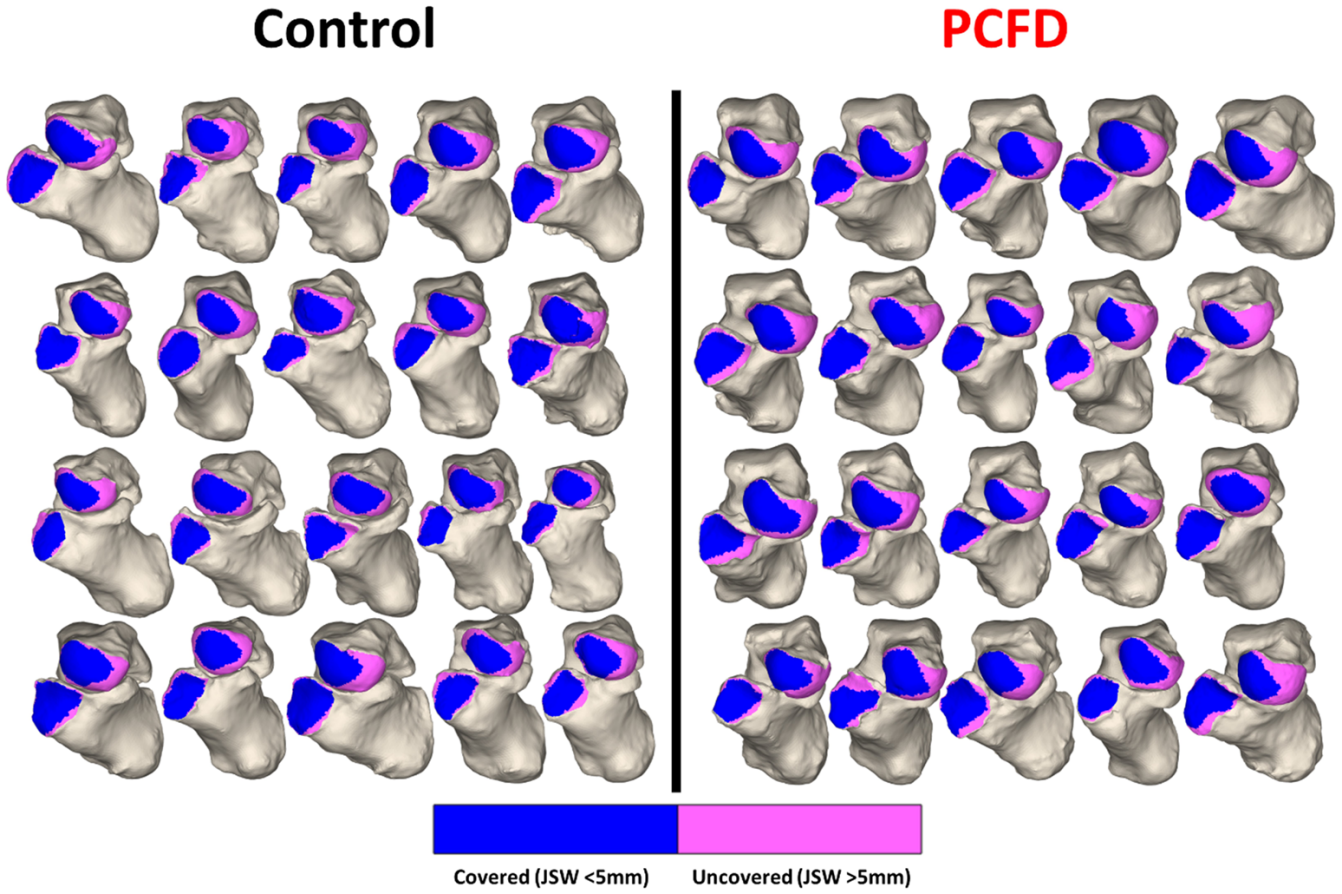
Chopart joint coverage maps for all control and Progressive Collapsing Foot Deformity feet. The navicular and cuboid are removed to show an anterior view of the talar head and calcaneocuboid facet. Pink indicates regions of uncoverage while blue represents regions of coverage.

Significant decreases in coverage were seen in all middle and medial regions of the talar head of PCFD patients in comparison to controls (Fig. 3). The largest decrease in coverage was seen in the plantar medial (− 79%, *p* = 0.003) and dorsal medial regions (− 77%, *p* = 0.00004). Coverage also decreased in PCFD for both the dorsal middle (− 23%, *p* = 0.001) and plantar middle (− 26% *p* = 0.003) regions. Corresponding increases in coverage were seen in both the dorsal lateral (+ 21%, *p* = 0.002) and plantar lateral (+ 30%, *p* = 0.0003) regions of the PCFD group. Overall coverage of the talar heads was similar among groups (*p* = 0.22). CMs for every joint are shown in Fig. 4. Percent coverage for each subregion is reported in Table 2.

**Table 2:** Means, medians, and standard deviations for 3-dimensional coverage maps, measured in percent coverage, and mean differences in the coverage percent in the Chopart joint when comparing patients with progressive collapsing foot deformity and controls, with *P* values for the comparisons.

Characteristic	Control, percent coverage			PCFD, percent coverage			Mean difference %	*P* values	
	Mean (%)	SD (%)	Median (%)	Mean (%)	SD (%)	Median (%)		T-test	MW test
Calcaneus	82.7	12.5	85.0	81.7	14.7	84.8	− 1.1	0.649	–
Medial	83.1	12.9	86.9	75.9	13.0	76.0	− 8.7	–	0.037
Plantar	87.9	10.2	92.1	77.4	16.1	83.7	− 11.9	–	0.006
Dorsal	78.1	11.9	79.2	81.5	15.8	86.2	4.3	0.079	–
Lateral	81.5	12.6	85.2	92.0	5.4	93.7	12.8	–	0.002
Talus	53.3	29.8	59.9	49.0	37.2	54.0	− 8.0	0.219	–
Dorsal									
Medial	21.5	16.5	15.9	5.0	8.7	1.4	− 76.5	–	0.00001
Middle	78.2	12.0	74.9	60.4	18.4	66.3	− 22.8	0.00121	–
Lateral	71.9	16.2	71.8	87.1	10.1	88.6	21.1	0.00154	–
Plantar									
Medial	13.6	11.9	8.8	2.9	4.5	0.8	− 78.6	–	0.0003
Middle	64.3	17.8	68.2	47.6	14.7	50.9	− 25.9	0.0032	–
Lateral	70.1	15.7	75.9	91.0	11.6	94.9	29.9	0.00004	–

*MW test* Mann–Whitney U test, *SD* standard deviation, *PCFD* progressive collapsing foot deformity.

**Figure 5:**
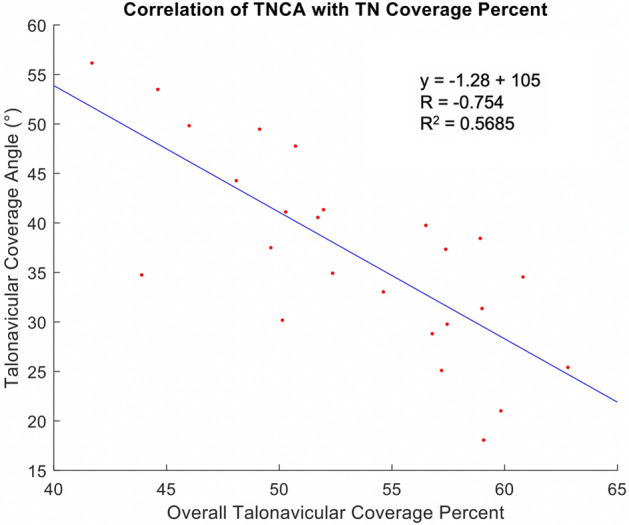
Correlation between the talonavicular three-dimensional (3D) coverage and the talonavicular coverage angle (TNCA). A moderate negative correlation was observed.

Changes in coverage were also observed in the calcaneocuboid facet. Coverage increased in the lateral region (+ 13%, *p* = 0.002) but not in the dorsal (+ 4%, *P* = 0.79) region of PCFD patients. A significant decrease in coverage was observed in both the plantar (− 12%, *p* = 0.006) and medial (− 9%, *p* = 0.037) regions. Changes in overall coverage of the calcaneocuboid facet were not significant (*p* = 0.649) when comparing the studied groups. TNCA had a mean of 31.52° (SD + − 6.78) in controls and 45.13 (SD + − 7.08) in PCFD patients (*p* < 0.001). Talonavicular coverage was negatively correlated (*r* = 0.75) and influenced (R^2^ = 0.57) by the TNCA (Fig. 5).

### Distance maps

**Figure 6:**
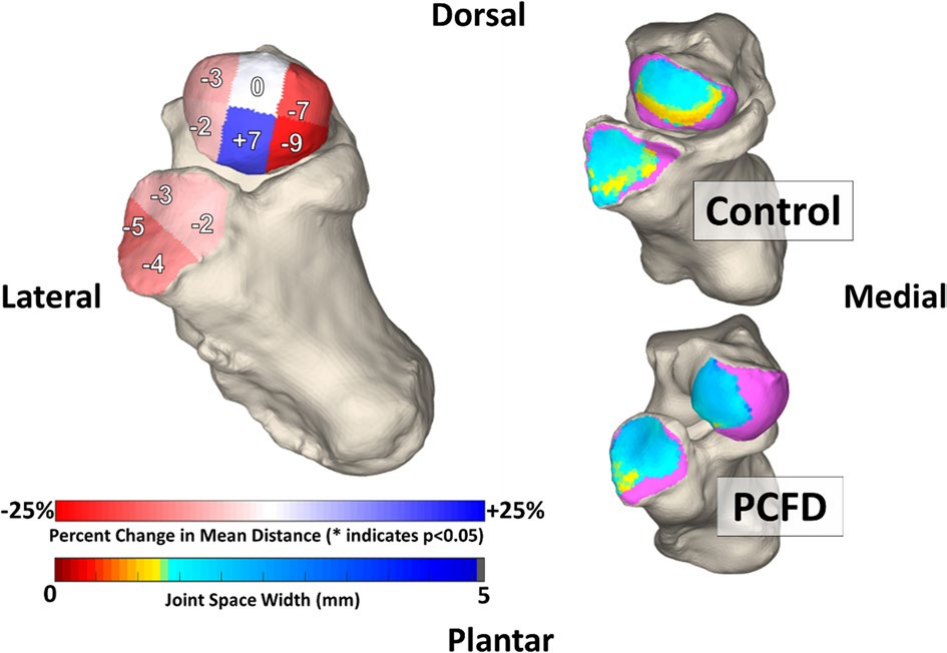
Average percent differences in distance for the 6 talar head and 4 calcaneocuboid facet regions. Red represents a decrease in decrease in distances from controls to Progressive Collapsing Foot Deformity patients while blue represents an increase.

**Figure 7:**
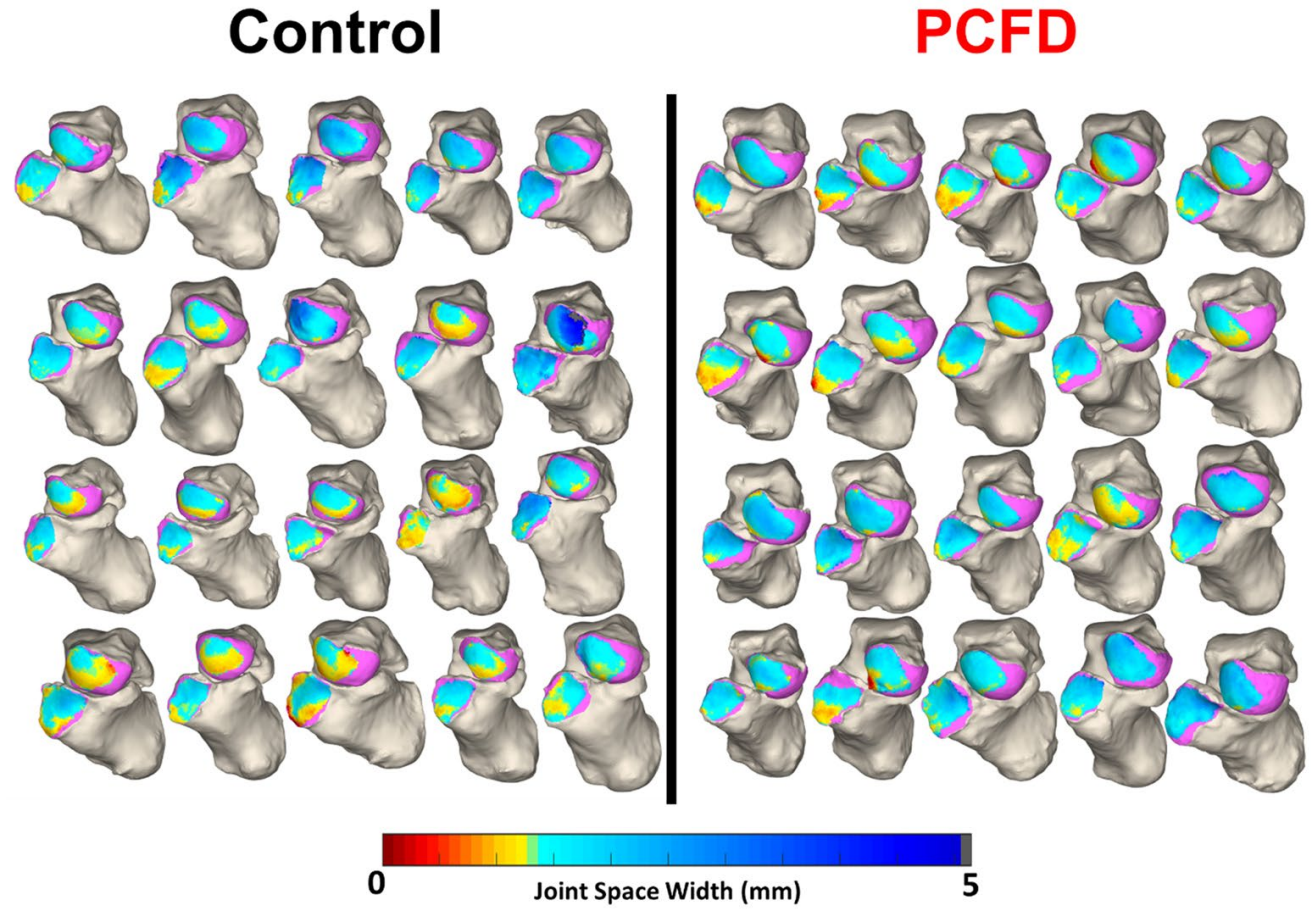
Chopart joint distance maps for all control and Progressive Collapsing Foot Deformity feet. The navicular and cuboid are removed to show an anterior view of the talar head and calcaneocuboid facet. Normal joint distances are shown in blue while distances over 5 mm are identified in gray and under 1.5 mm in green to yellow and under 1 mm in orange and red.

DM measurements for each joint (Fig. 6) are reported as means for each region in Table 3. There were no significant differences (ps > 0.224) in the mean distances for either the calcaneocuboid or the talonavicular articulation when comparing both groups. Additionally, there were no clear trends toward increased or decreased distances across cases (Fig. 7).

**Table 3:** Means and standard deviations for 3-dimensional distance maps, measured in millimeters, and mean differences in the distances in the Chopart joint when comparing patients with progressive collapsing foot deformity and controls, with *P* values for the comparisons.

Characteristic	Control, mm		PCFD, mm		Mean difference, %	*P* value
	Mean	SD	Mean	SD		
**Calcaneus**	1.73	0.40	1.67	0.32	− 3.6	0.256
Medial	1.91	0.37	1.86	0.30	− 2.3	0.688
Plantar	1.53	0.33	1.46	0.26	− 4.3	0.501
Dorsal	1.90	0.43	1.85	0.25	− 2.8	0.648
Lateral	1.62	0.32	1.53	0.24	− 5.3	0.362
**Talus**	1.53	0.43	1.50	0.57	− 1.9	0.654
Dorsal						
Medial	1.43	0.29	1.34	0.82	− 6.6	0.639
Middle	1.70	0.52	1.71	0.22	0.3	0.963
Lateral	1.81	0.49	1.76	0.25	− 2.9	0.680
Plantar						
Medial	1.38	0.43	1.26	0.97	− 8.6	0.648
Middle	1.39	0.27	1.48	0.18	6.6	0.224
Lateral	1.47	0.33	1.43	0.25	− 2.3	0.717

*SD* standard deviation, *PCFD* progressive collapsing foot deformity.

### Selection reliability

Selections of articular regions for the talus, calcaneus, cuboid, and navicular were compared across two trials taken at least 8 weeks apart. Average areas for each region are reported in Table 4 with intraclass correlations. Across all selections, the average difference was 9.4 ± 38.1 mm^2^ and the intraclass correlation coefficient for the areas was 0.9907.

**Table 4:** Intraclass correlation coefficient for selected regions, determined from area of regions.

Characteristic	Trial 1		Trial 2		Difference	
	Mean, mm^2^	SD, mm^2^	Mean, mm^2^	SD, mm^2^	MoD ± SD, mm^2^	ICC
Talus	946.7	155.9	947.6	144.9	− 0.9 ± 59.7	0.9604
Calcaneus	486.4	71.5	471.8	59.5	14.5 ± 45.8	0.8475
Navicular	569.0	95.3	558.2	91.9	10.9 ± 16.4	0.9893
Cuboid	447.0	55.1	433.7	55.7	13.3 ± 30.4	0.9036

*ICC* intraclass correlation coefficient, *MoD* mean of differences, *SD* standard deviation.

## Discussion

This study evaluated joint subluxation across the talonavicular and calcaneocuboid interfaces using distance and coverage mapping of full weightbearing CTs in patients with stage I flexible PCFD. Patients with PCFD presented with significant coverage changes in Chopart articular regions when compared to controls. The significant decrease in coverage on medial talar head and plantarmedial regions of the calcanelcuboid interface supported our primary hypothesis. Interestingly, in our cohort there were no significant differences in calcaneocuboid or talonavicular distances refuting our second hypothesis. Finally, selection of these regions proved reliable with an ICC > 0.99.

In patients with PCFD, subluxation occurred on both the talar head and the calcaneocuboid facet. Significant subluxation was noted on the medial side of the talar head in patients with PCFD, specially on its plantar aspects (plantar medial: − 79%; *p* = 0.003; plantar lateral: − 77%; *p* = 0.00004). Parallelly, the lateral regions on the talar head experienced an increase in coverage when compared to the controls (dorsal lateral: + 21%, *p* = 0.002; plantar lateral: + 30%, *p* = 0.0003). The changes in coverage on the plantar and dorsal subregions of the talar head were similar eliminating pure plantarflexion as the cause of this subluxation. This may indicate a tendency toward medial abduction and external rotation of the navicular as the root cause of these changes in coverage. These findings are in line with work by Louie et al., who was also able to identify a medial to lateral shift in coverage (*p* < 0.0001)^8^. However, Louie et al. found more pronounced plantar uncoverage than our cohort, potentially explained by differences in imaging acquisition (simulated weight-bearing CT and WBCT)^8^. Kitaoka et al. using cadaveric analysis, demonstrated a shift for a more central and dorsal contact distribution in PCFD patients^5^. Further, Malakoutikhah et al. observed a decrease in overall contact pressure and subluxation of the talonavicular joint when their finite model was collapsed^10^. These could contribute to the understanding that the deformity has a complex out of plane rotational component rather than a simple sagittal and axial movement^23,25,26^. Phan et al., using dual fluoroscopy, were able to observe similar behavior at the talonavicular joints of flatfoot patients, demonstrating increasing abduction (9.29; *p* = 0.003) and external rotational (11.17; *p* = 0.0032) in comparison to controls^9^.

On the calcaneocuboid facet, significant subluxation occurred at the plantar (− 12%, *p* = 0.006) and medial (− 9%, *p* = 0.037) subregions in patients with PFCD. Increases in coverage on the lateral and dorsal subregions occur, but only the lateral increase in coverage was significant (+ 13%, *p* = 0.002). Comparable comportment was observed by Phan et al. at the calcaneocuboid joints of flatfoot with higher external rotation movement (6.15, *p* = 0.351). The fact that PTS produces instability at the subtalar joint and the calcaneus also moves around the talus may explain why coverage changes were not as large on the calcaneal cuboid joint. Similarly, Wang et al. noticed lesser movement at the calcaneocuboid joint in comparison to the talonavicular and subtalar joints^27^. The study demonstrated 3.93°, 5.04° and 5.97° of dorsiflexion; 5.82°, 8.21°, and 15.46° of eversion; and 9.75°, 7.6°, and 4.99° of external rotation in normal feet during midstance in CC, talonavicular, and subtalar joints, respectively^27^.

Differences in overall coverage were not observed in either joint when comparing PCFD and control patients (*p* = 0.649) in our study. This is similar to what Louie et al. reported, finding similar overall coverage of the talus (62% vs. 56%) and navicular (98% vs. 92%) when comparing symptomatic flatfoot and neutral aligned subjects through CM. This is likely due to PTS increasing coverage and contact in some areas and decreasing by similar amounts in others thus providing a mean neutral value^7,12,23^. Another argument, raised by Louie et al., is that some of the PCFD can be secondary to a pediatric flatfoot and present dysplastic alterations to bone and joint that could create abnormal cartilaginous relations in subluxed areas^8,28,29^. The last possibility is that even in full weightbearing conditions, early PCFD may not experience true subluxation through the Chopart articulation. In this scenario hindfoot PTS, forefoot deformity, and ligamentous laxity cause changes through the midfoot that are within the compliance of highly mobile articulations.

This explanation is supported by the absence of differences seen among PCFD and controls in the overall and regional distance mapping (ps > 0.224). Since our sample is composed of flexible (stage 1) middle-age PCFD patients (mean 49.5 years old), early signs of joint degeneration (narrowing) would not be expected. In contrast to the subtalar joint where forces applied to the region are primarily vertical, making impingement (especially sinus tarsi) a valuable marker of topography, the tarsal joints present perpendicular to gravity resulting in increased potential for shearing and decreased potential for static extraarticular impingement^22,23^. A subluxation pattern might expect to see small decreases in distance on a side acting as a fulcrum to lever the opposing side out which would see an increase in distances. This pattern of DM changes were observed by Bernasconi et al. in their assessment of patients with asymptomatic pes planovalgus and controls, with decreases in distances superolaterally (− 20%, *p* = 0.097) and increases superomedially (+ 30.7% increase, *p* = 0.015) and inferomedialy (+ 45.1%, *p* = 0.001) talonavicular regions^30^. Similar to our study, no changes in distance were observed by this study at the calcaneocuboid joint^30^.

To account for potential differences in coverage derived from variance in selection, we evaluated the current gold standard of manual selection at two time points. Surface selections had a high reliability with an ICC greater than 0.99. The mean areas of the for the cuboid and talar head articulations were 433.7 and 947.6 mm^2^, respectively. Compared to the average area of the articular surfaces, the mean of differences between each trial averaged 9.4 ± 38.1 mm^2^. The average difference between the two selections was at most 10% of the overall area. These differences are negligible relative to the magnitude of differences seen in the overall talonavicular and calcaneocuboid joints; they are likely to average out over a population. However, increased reliability may be important to consider when looking at subregional analyses of individual cases where local variance in selection may impact results more dramatically. Therefore, automated methods are desirable to increase reliability before considering these results in the context of individual cases.

This study has several limitations. As a retrospective study, it could not evaluate the linear progression of the disease. Additionally, patients were not followed over time to identify changes secondary to PCFD. The study’s findings cannot be applied to class E (ankle valgus) and stage 2 (rigid) subjects which may involve later stage deformity. The matched control group consisted of a heterogeneous group of healthy volunteers. Although we observed statistically significant differences, previous sample calculations or power analysis were not performed. This could have underestimated the changes and contributed to similarities in overall coverage and distance mapping. Patient functional assessment was not executed, preventing correlation between symptoms and imaging findings. Finally, the use of WBCT and 3D coverage and distance mapping are still not widely accessible, decreasing the study’s reproducibility.

## Conclusion

In conclusion, our study results show that significant subluxation occurs on the medial region of the talar head and the plantar medial regions of the calcaneocuboid joint. To our knowledge, this is the first study to assess subluxation across the entire Chopart joint under full weightbearing conditions. These results provide a baseline to understand changes occurring at the transverse joint of PCFD patients, providing data that might help in disease management. Coverage and distance mapping provide objective information that could lead to earlier diagnosis and better assessment treatment impact when reestablishing joint interfaces. Future research is needed to increase reliability through automation and continue the search for more complete understanding of physiological bone congruence and changes associated with collapse and to halt articular degeneration in PCFD.

## Supplementary Information

Supplementary Information.

## Data Availability

According to the ICMJE data sharing police, core records will be shared through Mendeley Data and available upon request. Requests should be addressed to the corresponding author.
